# MRI Assessment of Associations between Brown Adipose Tissue and Cachexia in Murine Pancreatic Ductal Adenocarcinoma

**DOI:** 10.4172/2165-8048.1000301

**Published:** 2019-02-08

**Authors:** Yaqi Zhang, Su Hu, Junjie Shangguan, Liang Pan, Xin Zhou, Vahid Yaghmai, Yuri Velichko, Chunhong Hu, Jia Yang, Zhuoli Zhang

**Affiliations:** 1 Department of Radiology, Feinberg School of Medicine, Northwestern University, Chicago, IL, USA; 2 Department of Radiology, The First Affiliated Hospital of Soochow University, Suzhou, Jiangsu, China; 3 Department of Radiology, The Third Affiliated Hospital of Soochow University, Changzhou, Jiangsu, China; 4 Tianjin Key Laboratory of Cardiovascular Remodeling and Target Organ Injury, Pingjin Hospital, Tianjin, China; 5 Robert H. Lurie Comprehensive Cancer Center, Northwestern University, Chicago, IL, USA

**Keywords:** Pancreatic cancer, Brown adipose tissue, Cachexia, MR imaging

## Abstract

**Objective:**

As the major thermogenic tissue in body, the brown adipose tissue (BAT) was recently identified as an important factor to induce the rapid weight loss and malnutrition in malignancy. Current methods for detecting and quantifying brown adipose tissue (BAT) are in limited use. The aim of this study was to evaluate the changes of BAT tissue and its function in the development of pancreatic ductal adenocarcinoma (PDAC) by using magnetic resonance imaging (MRI).

**Methods:**

Ten-week-old female C57BL/6 mice were inoculated orthotopically with Pan02 tumor cells. R2* maps and two-point Dixon MRI were performed weekly for evaluation of BAT function and volume, respectively. The T2-weighted MRI was applied weekly for monitoring tumor growth. Meanwhile, the body weight was measured daily as another indication of malnutrition. The UCP1 levels in BAT and white adipose tissue (WAT) were assessed. The serum IL-6 was also measured as the biomarker of cancer-associated cachexia.

**Results:**

T2-weighted MRI indicated the rapid tumor growth from week 3 to week 5 after tumor cell inoculation. The water-fat separated MRI could clearly identify and quantify the BAT. The function and volume of BAT could be monitored by weekly MRI measurement in tumor-bearing mice. The total body weights of PDAC tumor-bearing mice were relatively stable, however, was significantly lower than that of control C57BL/6 mice.

**Conclusion:**

The results of this study demonstrated the feasibility of detection and quantification of BAT *in vivo* by MRI during the development of pancreatic cancer.

## Introduction

Pancreatic ductal adenocarcinoma (PDAC) is one of the most highly associated with wasting of peripheral tissues, metabolic syndrome that impairs quality of life, limits cancer therapy, leading to decreased survival rate [1]. It is reported that complex cancer-associated factors contribute to the metabolic dysfunction in PDAC patients, including circulative inflammation factors, anatomic factors (extrinsic compression from the tumor causing gastric outlet obstruction), and/or the adverse effects of chemotherapy [2]. Adipose wasting may also be caused by altered exocrine pancreatic function [3]. Although many studies have tried to reverse the symptom of cancer-associated cachexia, a complete reversal of wasting by using nutrition support or anti-cytokine treatment has not been achieved [2,4].

Brown adipose tissue (BAT) is adipose tissue that contains a high number of mitochondria within cells, making it an important thermogenic tissue for both basal and inducible energy expenditure. Primarily being found in human infants and young children, the presence of physiologically significant BAT was identified in normal adult human only less than a decade ago [5]. Besides its important role in obesity, BAT has been identified as a critical factor in metabolic dysfunction and cachexia in cancer patients due to its role in energy expenditure [1,6]. Therefore, tracking the changes of BAT is a promising biomarker for predicting the progression of PDAC and evaluating tumor response to therapy. However, the changes of BAT function and volume were not well-characterized because of limitations of non-invasive imaging methods for BAT.

Currently, ^18^F-FDG positron emission tomography-computed tomography (PET/CT) is the most commonly used platform for BAT imaging. However, some limitations still exist in the application of ^18^FFDG PET/CT. Firstly, the results of ^18^F-FDG-PET/CT are influenced by the injection amount and activity of radioactive tracers; Secondly, there is still no widely acceptable, optimized, and validated approach for data analysis and reporting. Although a guideline currently exists for BAT evaluation in FDG-PET/CT experiments, factors such as temperature and insulin level are difficult to normalize [7]. Besides, the high cost of ^18^F-FDG-PET/CT scans limits its utility in clinical work. On the other hand, magnetic resonance imaging (MRI) is a promising non-invasive technique with the advantages in lack of ionizing radiation, stability, versatility and lower-cost in identification and quantification of BAT [8,9]. Several MRI methods have been reported for analysis of BAT [9-11]. Compared with WAT, BAT is capillary-rich and multi-locular, indicating a higher ratio of water to fat. Therefore, the method of water-fat separated MRI, which is able to detect the fat fraction (FF) among different tissues, is an ideal method for identification of BAT. Additionally, Blood-oxygen-level-dependent (BOLD-MRI) can also been applied for BAT characterization because of the higher capillary content in BAT [8,10]. Due to the high mitochondrial density in BAT, BOLD-MRI could reflect the function of BAT, showing changes in the transverse signal decay rate (R2*). In infants and adolescents, R2* of supraclavicular BAT ranges from 39 to 84 s^−1^, and subcutaneous WAT ranges from 22 to 40 s^−1^, indicating R2* could be used as an indicator to evaluate the function of BAT [11,12]. Besides of the potential in measuring BAT, MRI is a well-established technique to detect and characterize neoplasia in patients, including early diagnosis and evaluation of treatment response [13]. Previously, the usefulness of T2-weighted imaging in detecting the growth of tumor has already been widely recognized [14].

In this study, the volume and function of BAT were evaluated in murine model of PDAC and compared with tumor development by MRI.

## Methods

### Cell culture

Panc02 cell line is derived from 3-amethylcholanthrene induced PDAC in C57BL/6 mice. Panc02 cells were cultured in RPMI 1640 medium (Life Technologies, Carlsbad, CA) supplemented with 10% fetal bovine serum (FBS, Sigma-Aldrich) and penicillin and streptomycin (100 IU/ml, Sigma-Aldrich). he cells were maintained in a humidified atmosphere of 5% CO_2_ at 37° C. Before tumor cell inoculation, cell viability was assessed using trypan blue staining (cell viability of >90% was confirmed).

### Animals

C57BL/6 female mice (10 weeks of age, weighting between 18 and 22 gm; Charles River, Wilmington, MA) were used in the study for establishing orthotopic pancreatic cancer models. Briefly, mice were anesthetized with 2% isoflurane in oxygen at a rate of 1 l/min. 1 × 10^5^ viable Panc02 cells were gently mixed with ice-cold Matrigel (Corning, NY, USA) at a ratio of 3:1 to produce a homogeneous suspension. Then the abdominal cavity was opened by a 1.5 cm longitudinal incision after local shaving and disinfection. 5 μL of Panc02 cell-matrigel mixture suspension was slowly injected into the tail of pancreas. The pancreas was placed back into the abdominal cavity, and the abdominal cavity was closed by a running two-layer silk suture. Postoperative status and wound healing were monitored every day for one week After one week, a visible nodule at the location of pancreatic tail was detected by magnetic resonance imaging in all mice (protocols described below), which indicated that the orthotopic pancreatic cancer models were established successfully. Body weight was measured and recorded daily. The mice were sacrificed on week 3, 4 and 5 respectively after Panc02 cell implantation. Our animal study was approved by the Institutional Animal Care and Use Committee (IACUC) of Northwestern University.

### Magnetic resonance imaging examination

MRI examinations were performed by using a 7.0 T small-animal MRI scanner (Clinscan, Bruker BioSpin, Ettlingen, Germany) with a commercial mouse coil (ClinScan, Bruker). One week after Panc02 cell implantation, tumors were detected on MRI in all the mice to confirm the successful establishment of orthotopic pancreatic cancer models. Mice were anesthetized by inhalation of a mixture of 2% isoflurane and oxygen at 1 l/min.

Firstly, tumor growth was monitored weekly. The MRI sequences and parameters were as follows:

(a) Axial T1-weighted imaging (T1WI): repetition time (TR)/echo time (TE)=630/20 ms; field of view (FOV)=27 mm ×30 mm; matrix size=122 × 192; slice thickness (ST)=0.7 mm; FA=90°;

(b) Axial T2-weighted imaging (T2WI): TR/ TE=158/40 ms; FOV=21 mm × 30 mm; matrix size=180 × 256; ST=0.8 mm; FA=180°;

(c) Coronal T2WI: TR/TE=2500/40 ms; FOV=26 mm × 36 mm; matrix size=142 × 192; ST=0.5 mm; FA=180°;

(d) Sagittal T2WI: TR/TE=2500/40 ms; FOV=24 mm × 30 mm; matrix size=154 × 192; ST=0.5 mm; FA=180°.

Then, MRI images of BAT were also performed weekly. Localization was performed using a T2-weighted sequence and was followed by shimming over the interscapular BAT. The MRI sequences for BAT were as follows:

(a) Sagittal T1-weighted imaging (T1WI; TR/TE=380/10 ms, voxel dimensions=0.156 × 0.156 × 0.6 mm^3^);

(b) Sagittal T2-weighted imaging (TR/TE=3000/66 ms, voxel dimensions=0.156 × 0.156 × 0.6 mm^3^);

(c) Sagittal Dixon imaging (TR=12 ms, TE=2/3.5 ms, voxel dimensions=0.09 × 0.09 × 0.6 mm^3^);

(d) Sagittal BOLD imaging (TR=30 ms; 4 echo times, TE 2.0 to 11.69 ms with 3.23 ms echo spacing; voxel dimensions=0.168 × 0.168 ×0 6 mm^3^).

Tumor size measurement was performed using ITK-SNAP (version 3.6.0, University of Pennsylvania) software, free-hand regions of interest (ROIs) were traced along the tumor margin on each slice of axial T2-weighted images containing an orthotopic tumor, and then the three-dimensional volume was calculated. R2* maps were created using a custom script in Matlab (The Math Works, Natick, MA, USA) based on BOLD imagings, and the R2* values of BAT were analyzed.

### H&E staining and immunohistochemistry

Pancreatic tumor, brown and white adipose tissue were collected separately, fixed by 10% formaldehyde and followed with 4 μm-section in thickness and stained with H&E staining. BAT and WAT sections from the same sample were processed with immunohistochemistry. Briefly, the antigen retrieval was performed in a water bath using citrate sodium buffer. After blocking for 1 h at room temperature in blocking buffer (5% goat serum, 2.5% BSA in 1 × PBS), sections were incubated with diluted anti-UCP1 antibody (Abcam, Cambridge, MA) overnight at 4° C. Then the sections were washed 3 times for 10 minutes with PBS and then incubated for 1 hour at room temperature with HRP-conjugated secondary antibody. Finally, immunostaining was detected with DAB and the slides were observed by digital camera.

### Quantitative qRT-PCR

BAT RNA was extracted and purified with RNeasy Lipid Tissue Mini kits (Qiagen Inc, Netherlands) and then reverse transcribed into cDNA with a Superscript IV VILO Master Mix (Invitrogen, Waltham, MA). qRT-PCR was performed using TaqMan Fast Advanced Master Mix (Thermo Fisher, Waltham, MA) and primer probes (Thermo Fisher, Waltham, MA). Thermogenic genes in BAT, uncoupling protein 1 (UCP1) and peroxisome proliferator-activated receptor γ (PPAR-γ) were compared between tumor-bearing mice and tumor naïve controls by qRT-PCR and normalized to tissue appropriate control genes (GAPDH).

### Enzyme-linked immunosorbent assay (ELISA)

The expression of IL-6 was detected by ELISA. The serum of tumorbearing mice and tumor-naïve mice were collected and determined by mouse IL-6 kit (R&D bioscience, Minneapolis, MN) according to the manufacturer’s protocols. The absorbance was measured at 450 nm with a micro plate reader.

### Statistics

For comparison between Pan02 tumor-bearing group and control group, data were assessed by Student’s t-test or analysis of variance (Prism 7.0, GraphPad Software, San Diego, CA). p<0.05 was used to assess statistical significance.

## Results

### Monitoring PDAC tumor development

**Figure 1:**
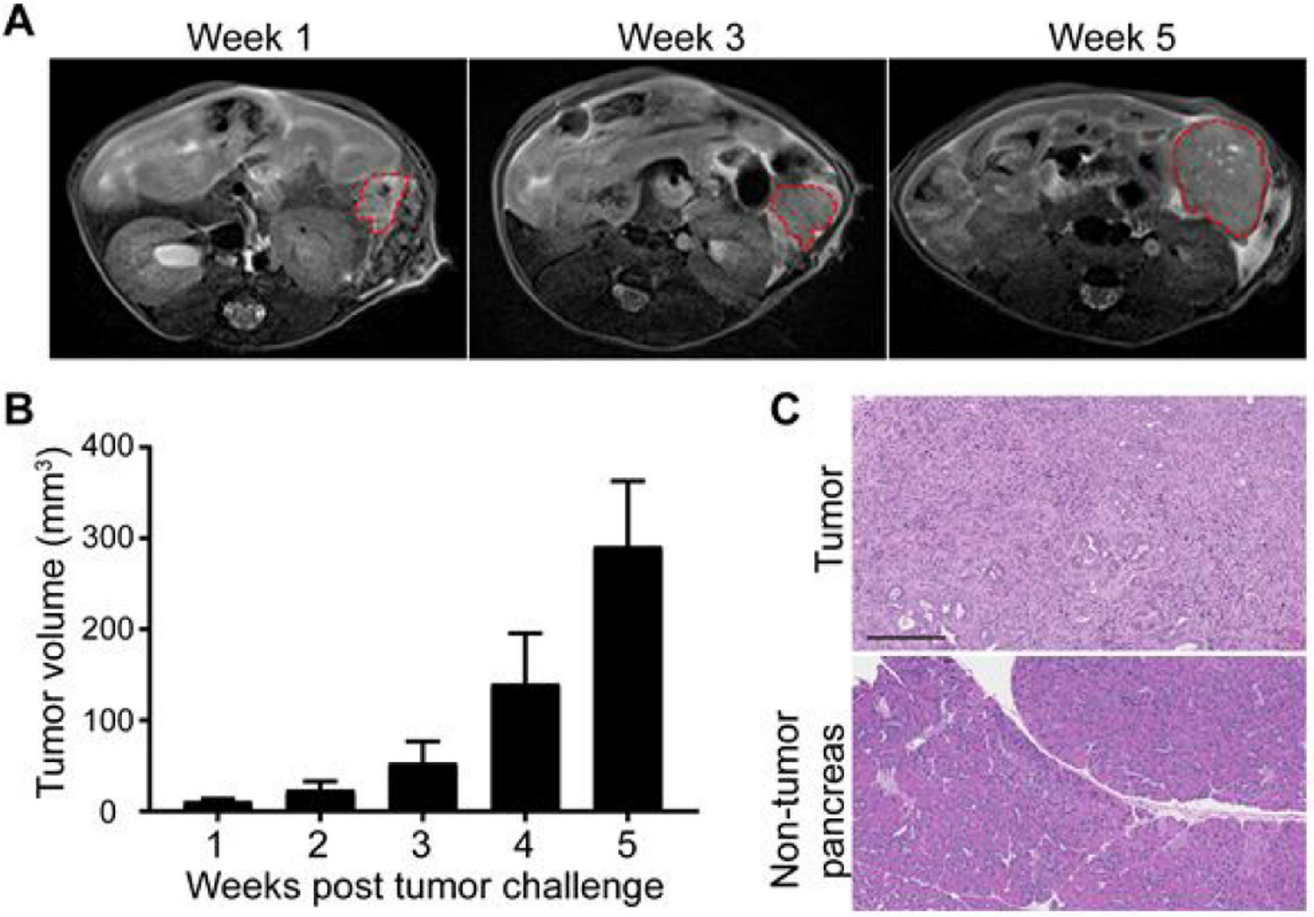
Monitoring tumor development after Panc02 cell implantation; (A) Representative T2-weighted MRI for pancreatic tumor on 1, 3 and 4 weeks after Panc02 cell implantation; (B) Quantitative result of tumor volume changes after Panc02 cell inoculation. Results were expressed as mean ± SEM; (C) H&E staining of PDAC tumor (upper panel) and adjacent non-tumor tissue (lower panel). Scale bar=200 μm.

Representative axial T2-weighted images of the pancreatic tumors at different time points after Panc02 cell implantation (Figure 1A). The average volumes of tumor from week 1 to week 5 were 11.01 ± 2.53, 23.8 ± 8.69, 53.21 ± 21.44, 140.20 ± 49.93 and 291.29 ± 63.03 mm^3^ respectively (Figure 1B). Histological examination showed that implanted tumor with pancreas duct-looking morphology and ragged infiltration compared with the adjacent pancreatic tissue, which confirmed the PDAC characteristics of our tumor model (Figure 1C).

### Body weight

**Figure 2:**
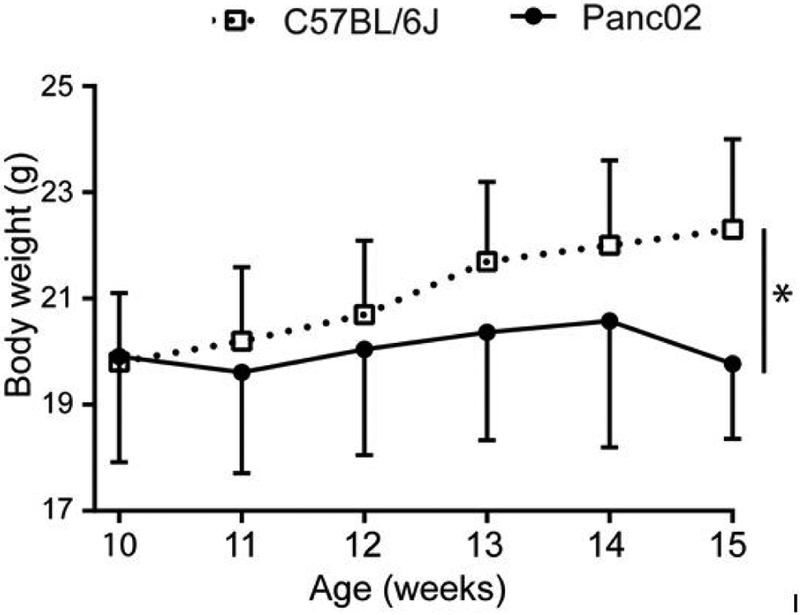
Body weight changes in tumor progression. Body weight changes compared with C57BL/6J WT mice. Results were expressed as mean ± SD.

Although the total body weight after Pan02 tumor inoculation remained stable, the tumor-bearing mice gained less weight compare with the C57BL/6 mice (Data from Jackson Lab) (Figure 2) [15].

### BAT function evaluation by MRI

**Figure 3:**
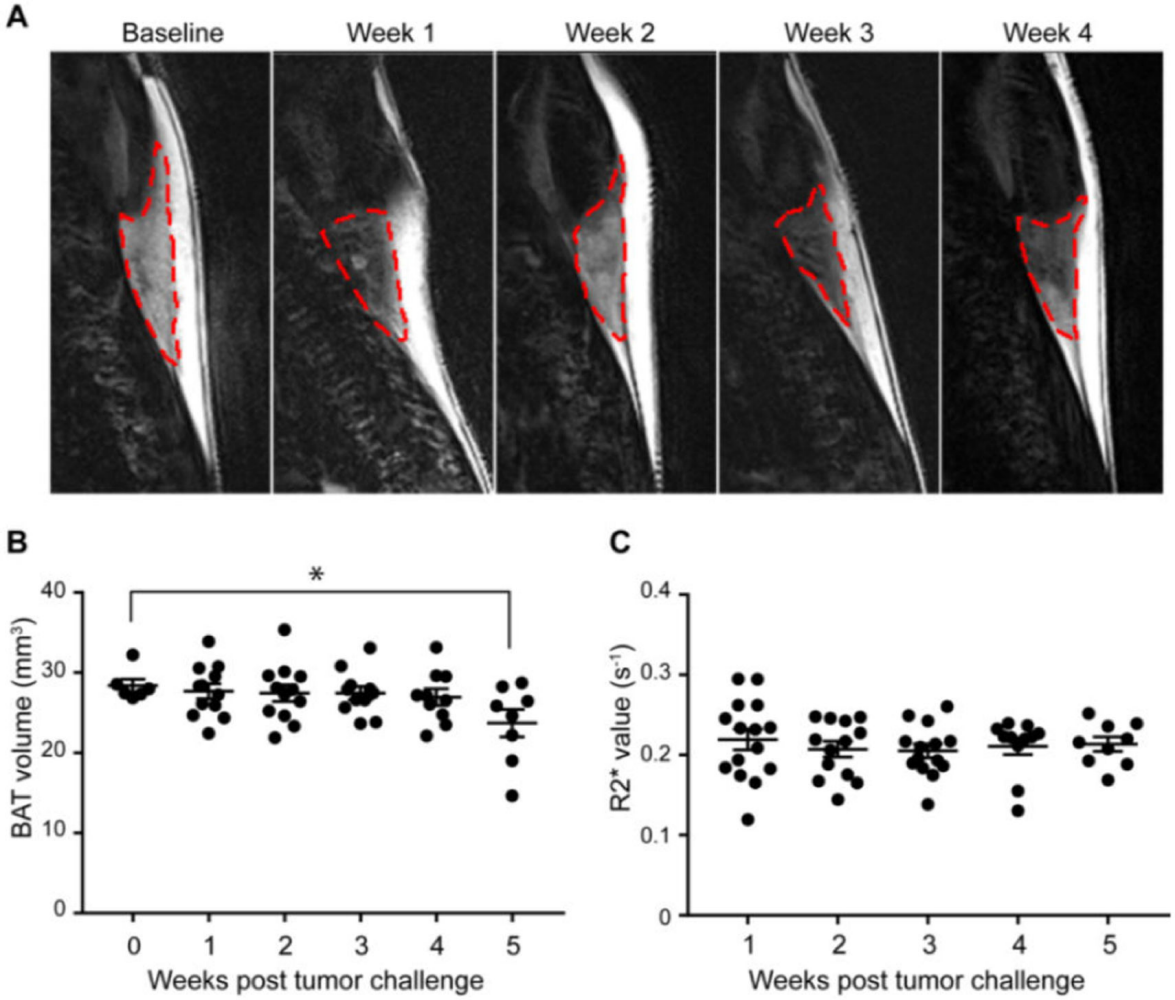
Changes of BAT during tumor growth; (A) Representative Dixon MRI (fat only) for BAT on 1-4 weeks after Panc02 cell inoculation. BAT areas are labeled with red dash line; (B) Changes in brown adipose volume based on ROI that determined by FF (* indicates to p<0.05); (C) R2* values in PDAC mice on week 1 to week 5 after Panc02 cell inoculation. Results were expressed as mean ± SEM.

In this study, the mice were scanned weekly for evaluating the changes in interscapular BAT after tumor cell orthotopic inoculation. The fat fraction image was calculated based on the result of Dixon MRI, and the ROIs (regions of interest) were selected based on the FF distribution in mice cervical region. As shown in Figure 3A, the BAT region could be clearly identified, as it is distinct from surrounding tissues, such as WAT and skeletal muscle. The volume of BAT was quantified by measuring the volume of ROIs while referring to the anatomical position based on T1 and T2 images. The volume of BAT was relatively stable in first 4 weeks, a significant decrease from 28.40 ± 0.73 to 23.71 ± 1.60 mm^3^ at week 5 (p=0.047, n 6-8 per group) (Figure 3B).

In order to detect the function of BAT after in PDAC model by MRI, the R2* values in BAT were measured based on BOLD sequences. As shown in Figure 3C, the average level of R2* shows no change with the increase in tumor size (Figure 3C).

### BAT mRNA expression and serum IL-6 ANALYSIS

**Figure 4:**
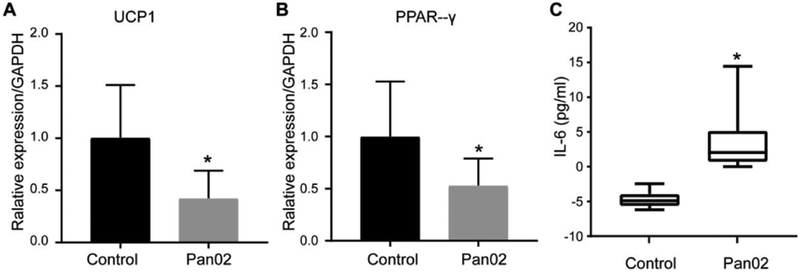
BAT mRNA expression and serum IL-6 concentration measurement; (A) qPCR analysis of UCP1 and PPCR-γ expression in BAT at 5 weeks after Panc02 cell inoculation and compared with age-matched control mice; (B,C) IL-6 levels in serum after Panc02 cell inoculation (* indicates to p<0.05). Results were expressed as mean ± SEM.

Recent study on the regulation of BAT development and function has led to the identification of key regulatory factors such as UCP1 and PPAR-γ. To further confirm the activity of BAT in tumor development, the mRNA expression levels of these two important functional proteins in BAT were measured. As shown in Figures 4A and 4B, both of their mRNA expression levels were significantly down regulated, a 57.55 ± 9.85 decrease of UCP1 expression (p<0.05, n=5-8 per group) and a 46.84 ± 8.54 decrease of PPAR-γ expression (p<0.05, n=5-8 per group), indicating the impairment of adipogenesis in PDAC model. IL-6, a prominent cachexia-associated factor in pancreatic cancer [16], which was remarkably increased in the serum of Pan02 tumor-bearing mice, whereas that of the tumor naive mice was below the detection limit (Figure 4C).

### Histologic characteristics and UCP1 protein expression in adipose tissue

**Figure 5:**
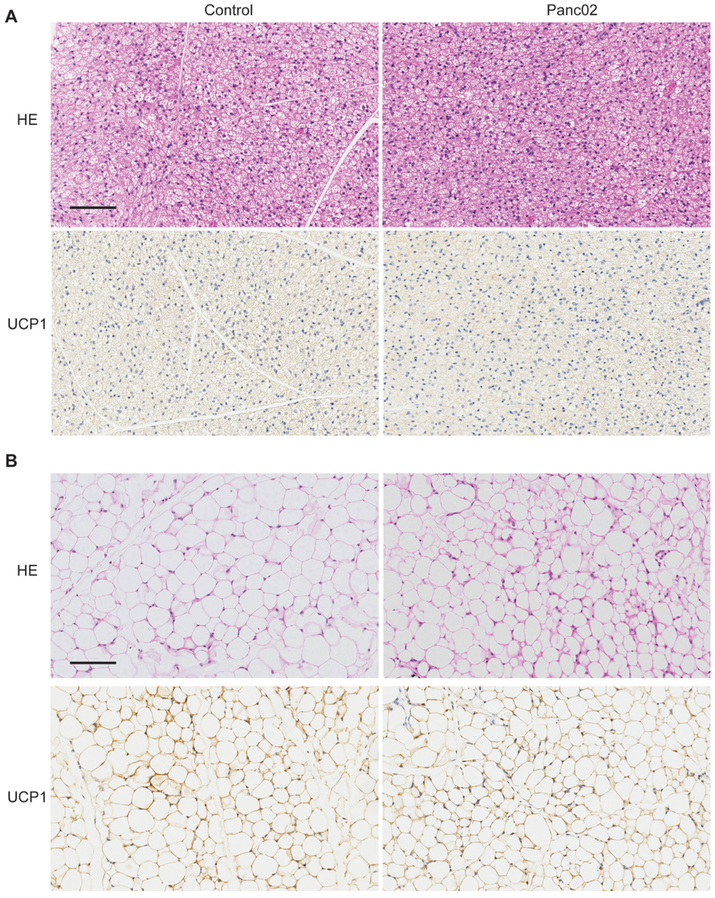
Histologic analysis of adipose tissue; (A) H&E staining and UCP1 protein in BAT on week 4 after Panc02 cell inoculation in tumor-bearing mice and age-matched control mice; (B) H&E staining and UCP1 protein expression in WAT on week 4 after Panc02 cell implantation in tumor-bearing mice and age-matched control mice. Scale bar=100 μm.

BAT from Pan02 tumor-bearing mice displayed smaller adipocytes containing less and/or smaller lipid droplets and showed a moderate reduction in UCP1 protein levels (Figure 5A). Histological analysis of inguinal WAT revealed the presence of smaller adipocytes with big nuclei and multilocular cytoplasm in Pan02 tumor-bearing mice, however, without changes of UCP1 protein levels (Figure 5B).

## Discussion

In this study we demonstrated the feasibility of utilizing Dixon-MRI for detection BAT tissue and evaluated the possibility to monitor the BAT thermogenic activity by BOLD-MRI in mouse PDAC model during tumor progression. The volume of BAT monitored by MRI was moderately decreased with the rapid tumor growth, while BOLD-MRI showed no changes of BAT at oxygen in the early phase of tumor. The tumor-bearing mice gained less weight in the development of tumor.

Cachexia is highly associated with cancers of the pancreas, esophagus, stomach, lung, liver and bowel [17]. In particular, PDAC presents a high penetrance of wasting, a process that seems to occur in earlier stages of tumor transformation [18]. The balances of nutrition and energy in PDAC patients are of great importance to their tolerance of treatment and overall survival. Although PDAC is one of the cancers most closely associated with cachexia, there are still limited experimental models available to quantify these changes. To this aim, we took advantage of Pan02 cells, a stable cell line derived from 3-amethylcholanthrene-induced PDAC in C57BL/6 mice [19]. While Greco and coworkers modeled cachexia by intraperitoneal injections of up to 10 million cells per mouse, thus exhibiting progressive weight loss within 2 weeks and subsequent animal death within 45 days [20]. We orthotopically inoculated only 1 × 10^5^ cells (the minimal amount necessary to consistently promote tumor growth) in order to promote a slower tumor growth in pancreas, thus recapitulating the cachexia features specifically associated with PDAC at early phases. This reduced cell number resulted in 50.13 ± 20.74 mm^3^ tumors at 3 weeks after injection (the time point where mice from Greco et al. already started to die). Pan02 tumor-bearing mice exhibited moderate weight loss within 1 week after tumor challenge, which may be due to operative itself. In the next 4 weeks, the total body weight after Pan02 tumor inoculation remain stable, but the tumor-bearing mice gained less weight compare with the C57BL/6 mice.

BAT plays a key role in thermogenesis and energy balance and potentially contributes to the physiologic perturbations associated with cachexia [21]. Since MRI does not use harmful ionizing radiation, as opposed to PET/CT, it is more suitable for studying BAT activity in longitudinal studies on healthy subjects. However, few studies have focused on using MR imaging technologies to detect and monitor the changes of BAT during tumor progression. We demonstrated the feasibility of measuring BAT volume *in vivo* using routine MRI sequences. In our results, BAT volume of Pan02 tumor-mice was slightly decreased in the first 4 weeks after tumor inoculation, and moderately decreased at 5 weeks, which confirmed the early malnutrition in PDAC. However, the R2* and R2* value showed no change during the development of tumor, despite the possibility to use BOLD-MRI for the quantification of BAT activity in mice as previously described [11]. Furthermore, in accordance with previous reports [22,23], expression of 2 genes particularly associated with BAT thermogenesis, UCP1 and PPAR-γ were decreased, which indicating the impaired function of BAT in PDAC model. Importantly, histological analysis of the adipose tissues showed significant morphologic alterations and decreased expression of UCP1 in Pan02 tumor-bearing mice. It is well known that elevated levels of IL-6 are associated with increased cachexia and decreased survival in pancreatic cancer patients [20,24]. Accordingly, we also detected an upregulation of IL-6 in Pan02 tumor-bearing mice.

The current study had several limitations. For instance, there were no significant differences of R2* with tumor growth, which are unexpected results. We are developing a new gas-challenge BOLD MRI sequence to monitor BAT function in PDAC mouse model. Furthermore, the data set presented here is the focus on evaluating the BAT changes in early PDAC mice (in 5 weeks after tumor challenge). Further investigations evaluating the effect of advanced stage of cancer on cachexia are required. In addition, recent studies on pancreatic cancer cachexia modeled in mice suggested that Pdx1Cre; KrasG12D; Tp53R172H (KPC) congenic allografts recapitulate key features of PDAC disease process and induce a wide array of cachexia features [3,22]. Further study use of KPC mouse model is needed.

In summary, we demonstrated the feasibility of detection and quantification of BAT *in vivo* by MRI during the development of pancreatic cancer.

## References

[1] Petruzzelli M, Wagner EF (2016) Mechanisms of metabolic dysfunction in cancer-associated cachexia. Genes Dev 30: 489–501. 26944676 10.1101/gad.276733.115 PMC4782044

[2] Nemer L, Krishna SG, Shah ZK, Conwell DL, Cruz-Monserrate Z, (2017) Predictors of pancreatic cancer-associated weight loss and nutritional interventions. Pancreas 46: 1152–1157. 28902785 10.1097/MPA.0000000000000898 PMC5679236

[3] Danai LV, Babic A, Rosenthal MH, Dennstedt EA, Muir A, (2018) Altered exocrine function can drive adipose wasting in early pancreatic cancer. Nature 558: 600–604. 29925948 10.1038/s41586-018-0235-7 PMC6112987

[4] Penna F, Minero VG, Costamagna D, Bonelli G, Baccino FM, (2010) Anti-cytokine strategies for the treatment of cancer-related anorexia and cachexia. Expert Opin Biol Ther 10: 1241–1250. 20594117 10.1517/14712598.2010.503773

[5] Cypess AM, Fehman S, Williams G, Tal I, Rodman D, (2009) Identification and importance of brown adipose tissue in adult humans. N Engl J Med 360: 1509–1517. 19357406 10.1056/NEJMoa0810780 PMC2859951

[6] Petruzzelli M, Schweiger M, Schreiber R, Campos RO, Tsoli M, (2014) A switch from white to brown fat increases energy expenditure in cancer-associated cachexia. Cell Metab 20: 433–447. 25043816 10.1016/j.cmet.2014.06.011

[7] Bao J, Cui X, Cai S, Zhong J, Cai C, (2013) Brown adipose tissue mapping in rats with combined intermolecular double-quantum coherence and dixon water-fat MRI. NMR Biomed 26: 1663–1671. 23852574 10.1002/nbm.3000

[8] Hu HH, Smith DL, Nayak KS, Goran MI, Nagy TR (2010) Identification of brown adipose tissue in mice with fat-water Ideal-MRI. J Magn Reson Imaging 31: 1195–1202. 20432356 10.1002/jmri.22162 PMC2924147

[9] Holstila M, Pesola M, Saari T, Koskensalo K, Raiko J, (2017) MR signal-fat-fraction analysis and T2* weighted imaging measure BAT reliably on humans without cold exposure. Metabolism 70: 23–30. 28403942 10.1016/j.metabol.2017.02.001

[10] Rasmussen JM, Entringer S, Nguyen A, van Erp TG, Burns J, (2013) Brown adipose tissue quantification in human neonates using water-fat separated MRI. PLoS One 8: e77907. 24205024 10.1371/journal.pone.0077907 PMC3813555

[11] Khanna A, Branca RT (2012) Detecting brown adipose tissue activity with BOLD MRI in mice. Magn Reson Med 68: 1285–1290. 22231619 10.1002/mrm.24118 PMC3326213

[12] Hu HH, Hines CD, Smith DL, Reeder SB (2012) Variations in T(2)* and fat content of murine brown and white adipose tissues by chemical-shift MRI. Magn Reson Imaging 30: 323–329. 22244539 10.1016/j.mri.2011.12.004 PMC3288644

[13] Miller FH, Rini NJ, Keppke AL (2006) MRI of adenocarcinoma of the pancreas. AJR Am J Roentgenol 187: W365–W374. 16985107 10.2214/AJR.05.0875

[14] Scheidler J, Heuck AF, Steinborn M, Kimmig R, MF R (1998) Parametrial invasion in cervical carcinoma: Evaluation of detection at MR imaging with fat suppression. Radiology 206: 125–129. 9423661 10.1148/radiology.206.1.9423661

[15] The Jackson Laborator (2018), Body weight information for C57BL/6J (000664). Jax Home.

[16] Martignoni ME, Kunze P, Hildebrandt W, Kunzli B, Berberat P, (2005) Role of mononuclear cells and inflammatory cytokines in pancreatic cancer-related cachexia. Clin Cancer Res 11: 5802–5808. 16115919 10.1158/1078-0432.CCR-05-0185

[17] Baracos VE, Martin L, Korc M, Guttridge DC, Fearon KCH (2018) Cancer-associated cachexia. Nat Rev Dis Primers 4: 17105. 29345251 10.1038/nrdp.2017.105

[18] Mayers JR, Wu C, Clish CB, Kraft P, Torrence ME, (2014) Elevation of circulating branched-chain amino acids is an early event in human pancreatic adenocarcinoma development. Nat Med 20: 1193–1198. 25261994 10.1038/nm.3686 PMC4191991

[19] Corbett TH, Roberts BJ, Leopold WR, Peckham JC, Wilkoff LJ, (1984) Induction and chemotherapeutic response of two transplantable ductal adenocarcinomas of the pancreas in c57bl/6 mice. Cancer Res 44: 717–726. 6692374

[20] Greco SH, Tomkotter L, Vahle AK, Rokosh R, Avanzi A, (2015) Tgf-beta blockade reduces mortality and metabolic changes in a validated murine model of pancreatic cancer cachexia. PLoS One 10: eO 132786. 10.1371/journal.pone.0132786 PMC4501823 26172047

[21] Tsoli M, Moore M, Burg D, Painter A, Taylor R, (2012) Activation of thermogenesis in brown adipose tissue and dysregulated lipid metabolism associated with cancer cachexia in mice. Cancer Res 72: 4372–4382. 22719069 10.1158/0008-5472.CAN-11-3536

[22] Michaelis KA, Zhu X, Burfeind KG, Krasnow SM, Levasseur PR, (2017) Establishment and characterization of a novel murine model of pancreatic cancer cachexia. J Cachexia Sarcopenia Muscle 8: 824–838. 28730707 10.1002/jcsm.12225 PMC5659050

[23] Lasar D, Rosenwald M, Kiehlmann E, Balaz M, Tall B, (2018) Peroxisome proliferator activated receptor gamma controls mature brown adipocyte inducibility through glycerol kinase. Cell Rep 22: 760–773. 29346772 10.1016/j.celrep.2017.12.067

[24] Mitsunaga S, Ikeda M, Shimizu S, Ohno I, Furuse J, (2013) Serum levels of IL-6 and IL-lbeta can predict the efficacy of gemcitabine in patients with advanced pancreatic cancer. Br J Cancer 108: 2063–2069. 23591198 10.1038/bjc.2013.174 PMC3670479

